# Lung cancer: a new frontier for microbiome research and clinical translation

**DOI:** 10.3332/ecancer.2018.866

**Published:** 2018-09-05

**Authors:** Luis AJ Mur, Sharon A Huws, Simon JS Cameron, Paul D Lewis, Keir E Lewis

**Affiliations:** 1 Institute of Biological, Environmental and Rural Sciences, Aberystwyth University, Penglais Campus, Aberystwyth SY23 2DA, UK; 2 Institute for Global Food Security, School of Biological Sciences, Medical Biology Centre, Queen’s University Belfast, 97 Lisburn Road, Belfast BT9 7BL, UK; 3 Division of Computational and Systems Medicine, Department of Surgery and Cancer, Imperial College London, Charing Cross Hospital Campus, London W6 8RD, UK; 4 College of Medicine, Swansea University, Swansea SA2 8PP, UK; 5 Respiratory Unit, Prince Philip Hospital, Llanelli SA14 8QF, UK; 6 School of Medicine, University of Wales Swansea, Swansea SA2 8PP, UK

**Keywords:** lung cancer, microbiome, Granulicatella, ATP

## Abstract

The lung microbiome has been shown to reflect a range of pulmonary diseases—for example: asthma, chronic obstructive pulmonary disease (COPD) and cystic fibrosis. Studies have now begun to show microbiological changes in the lung that correlate with lung cancer (LC) which could provide new insights into lung carcinogenesis and new biomarkers for disease screening. Clinical studies have suggested that infections with tuberculosis or pneumonia increased the risk of LC possibly through inflammatory or immunological changes. These have now been superseded by genomic-based microbiome sequencing studies based on bronchoalveolar lavage, sputum or saliva samples. Although some discrepancies exist, many have suggested changes in particular bacterial genera in LC samples particularly, *Granulicatella, Streptococcus and Veillonella. Granulicatella* is of particular interest, as it appeared to show LC stage-specific increases in abundance. We propose that these microbial community changes are likely to reflect biochemical changes in the LC lung, linked to an increase in anaerobic environmental niches and altered pyridoxal/polyamine/nitrogenous metabolism to which *Granulicatella* could be particularly responsive. These are clearly preliminary observations and many more expansive studies are required to develop our understanding of the LC microbiome.

## The lung microbiome

The concept that humans have ‘another genome’ in the associated microbiome is now firmly established [1]. Although not ignored, the lung microbiome has received less attention compared with the gut which could be a relic of the historical view that the normal lung is free from bacteria [2]. In fact, with typical air exchanges as expressed in the respiratory minute volume of 6 L (0.5 L × 12 breaths/minute), the upper and lower respiratory tracts are major sites of microbial exchanges with the environment [3]. Indeed, it is believed that between 1,500 and 14,000 microbes are inhaled each hour and the composition of the healthy lung microbiome is likely to reflect relative immigration, elimination through mechanisms such as coughing and microbial growth [4]. Within the healthy lung, epithelial layers are covered with only a thin mucosal layer with only <100 mL of mucus being produced per day; but are rich in lipid-rich surfactant to prevent alveolar collapse [5]. Therefore, the lung is a relatively low-nutrient environment for microbes. The lung microbiome is also distinctive to that of the upper respiratory tract [6] which displays greater similarities to that found in the stomach [7], although there are distinctive oral microbiomes reflecting discrete habitats [8].

The work of Hilty *et al* [6] represents a foundational study in lung microbiomics. Based on bronchial lavages of 43 patients, containing healthy individuals and those suffering from either chronic obstructive pulmonary disease (COPD) or asthma they found that seven genera dominated the lung, namely, *Corynebacterium, Prevotella, Staphylococcus, Streptococcus, Veilonella, Haemophilus* and* Neisseria* as defined by 16S rRNA gene sequencing. The lung microbiome is now a well-established focus of research into asthma, COPD and cystic fibrosis (CF). Nonetheless, there remain some technical questions regarding how to effectively sample the lung microbiome. Many studies have concentrated on bronchial lavages that are saline washes of parts of the lung obtained using a bronchoscope (e.g., [6, 7, 9]). This minimises the risk of contamination from the upper respiratory tract but clearly causes considerable patient discomfort. Sputum represents an alternative sampling route which, although representing an increased risk of contamination, can provide important insights into the lung microbiome [4].

Most likely due to its poor nutrient status, the number of bacteria is relatively low in healthy lungs but can increase markedly with respiratory disease [4]. This can reflect changes in pH, oxygen tension, temperature but perhaps most importantly changes in the adaptive and innate defence responses often linked to inflammatory events [10]. These are often linked to a substantial increase in the production of mucus which can represent an increased microbial nutrient source and this can be supplemented by free adenosine triphosphate [11] to encourage the growth of certain microbial species [4]. Considerations of which microbial changes occur during a range of lung pathologies have been the focus of many recent studies, which feature progressive worsening of chronic disease with occasional marked symptom exacerbations.

In asthma, transient incursions from *Streptococcus* are good predictors of asthma [12]. With CF disproportionately affects lung function due to a substantial build-up mucus, inflammation and microbial changes particularly *Staphylococcus aureus, Haemophilus influenzae* and *Pseudomonas aeruginosa* [13]. Microbiomic studies have suggested that some fermentative anaerobes are important to CF exacerbations [14]. COPD is a progressive disease associated with increasing shortness of breath and sputum production. Although the microbiome does not appear to change during the early stages of COPD as the disease advances there appear to be shifts towards genera which include pathogenic strains of *Streptococcus*, *Pseudomonas* and* Haemophilus* [15, 16]. The presence of pathogenic bacterial species is likely to be most significant during COPD exacerbations [17].

## Microbial associations with lung cancer

Lung cancer (LC) remains a major global challenge, still responsible for 1.3 million deaths each year due to poor survival rates post-diagnosis. These relatively poor survival rates, compared to other cancers, are primarily a result of the late detection of a malignancy, where the success rates of clinical interventions are significantly reduced. LC exists in many histological types, each linked to various aetiology, developmental patterns and prognoses [18]. Whilst a role for the microbiome in LC does not seem to be immediately obvious compared to diseases such as CF or COPD, inflammatory responses are also a feature of LC [19] and this must be a starting point for considering a role for the microbiome.

Smoking is one of the prime risk factors in cancer and the associated carcinogens can readily form DNA adducts to promote genomic mutation. Equally, smoking can lead to chronic inflammation [20] and this may be the source of possibly 25% of all cancers [21, 22]. Mechanistically, this may arise from leukocytes producing reactive oxygen species, nitric oxide, metalloproteinases, interleukins and interferons, which could contribute to carcinogenesis by promoting increased genomic instability [23] Furthermore, cyclooxygenase (COX)-derived prostaglandin E2 can induce both tumour growth and metastasis. Given this, it has been shown that COX-inhibiting aspirin could suppress the development of LC although considerable heterogeneity amongst different sub-populations was observed [24].

If LC can arise from proinflammatory events, could chronic microbial infections could contribute to such as LC or respond to associated changes in the malignant lung? Such a link is apparently strong for a range of other cancers, especially for *Helicobacter pylori* and gastric cancer [25]. Here, the bacterial production of the *CagA* and some other 27–31 genes in a 40 kb gene cluster known as a pathogenicity island elicits a pronounced inflammatory response [26]. Other examples of bacterial infections linked to cancer are known [27] and are also referred to in other reviews in this edition of eCancer. In addition, the literature continues to provide some good indications that chronic lung diseases, such as tuberculosis (TB), as well as acute infection with pneumonia could be linked to lung carcinogenesis.

TB is the world’s leading cause of death from an infectious disease and been linked to around 1.4 million deaths in 2015 [28]. The causal agent is *Mycobacterium tuberculosis* (*M. tuberculosis*), which the following inhalation will infect and replicate within endosomes occurring in alveolar macrophages. Attempts by the macrophage to neutralise the bacterium are thwarted by the mycolic acid-based bacterial capsule. Once established, infected macrophages, T lymphocytes, B-lymphocytes and fibroblasts form a granuloma. More macrophages are recruited and fused to form multinucleated giant cells or lipid-rich foamy cells in the maturing granuloma. Crucially, many of these features are pro-inflammatory, and indeed, Tumour Necrosis Factor (TNF-a) and interferon (IFN-g) play an important role in driving granuloma formation. The granuloma, however, could be considered a failed form of host defence as *M. tuberculosis* may lie in a latent form for many years. The granuloma will eventually disintegrate allowing the bacteria to spread and form new lesions. However, about 90% of people infected with *M. tuberculosis* have asymptomatic, latent *M. tuberculosis* infections which very rarely activate to form an active disease [29].

Given the potential for TB infections to be foci for inflammation a possible link with LC was suspected but only recently has been supported by clinical studies [30]. A recent Danish study compared the cancer risk in TB patients during 1978–2011 with the general population and noted a significantly increased risk of LC [48]. Such an increased risk of LC in TB patients was also suggested in studies from Taiwan [31] and South Korea [32]. Histological studies have identified when LC and TB were coincident or where tumours formed from areas of pulmonary fibrosis linked to older sites of TB infection (TB scars) or from tuberculous cavities [33]. Extensive fibrosis may be associated with recurring TB infections and could decrease the clearance of lung lymph and lymph-associated particles. These have been linked to suppression of p21 to promote exit from G^o^ in the cell cycle, increased expression of Bcl-2 will reduce apoptosis leading to genomic instability and finally, pro-inflammatory activation of cyclooxygenase 2 (COX-2) to produce eicosanoids which encourage angiogenesis [34].

Such observations are highly suggestive but a causal link is still uncertain as the immunosuppressive status seen with LC and its treatment could reactivate TB [35] so that this could bias any observations. Also, the possible role of latent TB infections, by far the most common form and carcinogenesis in the lung is unknown. Furthermore, it is unknown if inflammatory events linked to other chronic infection events could also be associated with LC. Thus, the SYNERGY project collected data from Canadian and EU patients with a chronic bronchitis, emphysema, TB and pneumonia which were tested for association with LC [36]. Interestingly, this study found no relationship between TB and LC. In contrast, a positive relationship was observed between LC and pneumonia, if it was diagnosed 2 years previously. However, no such association was seen in the female group which could reflect lower degrees of smoking. The strongest relationship was observed in patients where chronic bronchitis was present with either emphysema or pneumonia. In another population-based study in Taiwan, the incidence rate of LC was significantly higher in pneumococcal pneumonia patients than in controls with a striking hazard ratio of 4.24 [37].

## The lung cancer microbiome

Given the observations of possible associations with microbial, pulmonary disease and LC, it is clear that microbiome investigations could provide some novel insights and potential biomarkers [38]. An initial retrospective study of bronchial aspirations collected by bronchoscopic endoscopy (*n* = 216), reported increases in potential pathogens of Gram-negative bacilli; including *H. influenzae, Enterobacter* sp., *Escherichia coli* as well as the Gram-positive Mycobacteria [39]. Metataxonomic 16S rRNA gene sequencing approaches were used by Lee *et al* [40] and 28 patients (20 LC, 8 benign biomass controls), again using bronchoalveolar lavage fluid (BALF). Two genera (*Veillonella* and *Megasphaera*) were significantly increased in LC patients and together could predict LC with an accuracy of 0.888 (as indicated with an area under the curve) [40]. In the following year, a paper was published on the salivary microbiome in patients (*n* = 10 in each category) with squamous cell carcinoma, adenocarcinoma and controls using targeted QPCR and 16S rRNA gene sequencing-based metataxonomics. They demonstrated that the salivary microbiome was different in the saliva of LC patients, particularly with respect to *Capnocytophaga*, *Selenemonas*, *Veillonella* and *Neisseria*. Using targeted QPCR they confirmed that *Capnocytophaga* and *Veillonella* were more abundant in LC patients, whilst *Neisseria* was conversely less abundant, suggesting their potential use as a bacterial biomarker. Of particular interest is the fact that in combination these could distinguish squamous cell carcinoma or adenocarcinoma from controls with significant but varying accuracies [41].

In another publication, the LC microbiome was investigated using paired samples of bronchial brushings from 24 LC patients one from the cancerous site, paired with a nearby noncancerous site and 18 from healthy controls from bronchoscopies [42]. They showed that the LC site exhibited decreased microbial diversity. However, the LC sites were enriched in *Streptococcus* compared to the controls which could be used to predict LC. Interestingly, this pair-sampling approach in LC patients suggested that the cancerous and non-cancerous microbiome were not very dissimilar which would argue for the LC microbiome reflecting wide-ranging changes in the lung [42].

**Figure 1.**
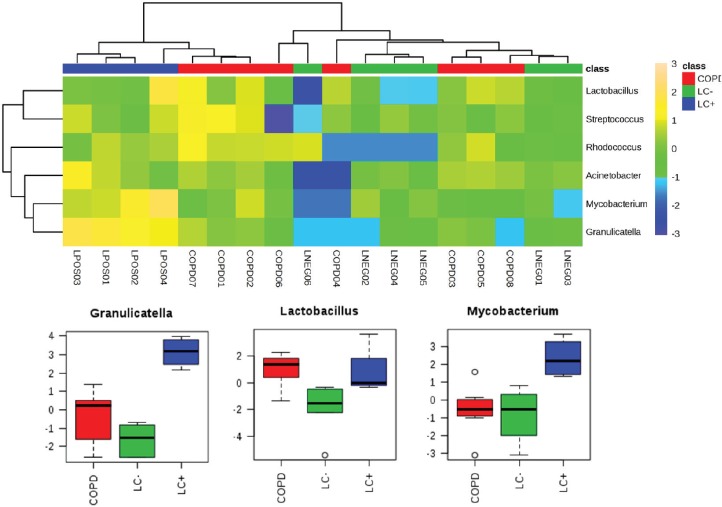
Significantly significant features of the LC microbiome which are observed in the sputum from patients with LC or COPD. We have previously published metagenomics studies of LC and COPD patients [44, 48]. Working at the level of ‘genus’, those showing significant differences between the microbiomes from LC (LC+) patients to patients referred to respiratory clinics, which were not found to have LC (LC−) or to COPD patients (further experimental details are found in [44, 48]), were extracted and compared using a heat map. Three genera showed significant increases in the LC+ category and these are presented in box and whisker format to depict log10 microbial abundance data.

Household pollution linked to burning coal may also be an LC risk factor which could be linked to the production of polycyclic aromatic hydrocarbons. A microbiomic component was tested in a never-smoked population of women from China with LC. This small-scale study revealed significant increases in *Granulicatella*, *Abiotrophia* and *Streptococcus* within sputum compared with the sputum of healthy controls [43]. Our own studies into the lung microbiome applied a full metagenomic approach to allow more insights into the LC microbiome in sputum [44]. Although this was a pilot study (*n* = 10), we noted increases in the range of species and in particular, *Granulicatella adiacens* which aligned with the study of Hosgood *et al* [43] . The increases in *G. adiacens* appeared to be particularly important as these correlated with the LC stage. As we have recently also published a metagenomic study of the sputum of COPD patients [16], we could compare the two pathologies compared to controls (Figure 1). To ease comparison with 16S rRNA amplicon-based studies, the heat map in Figure 1 focuses only significant changes at the level of genus. This indicated the increases in *Granulicatella* to be specific to LC (*P =* < 0.001, FDR 0.0004)*,* but there were also significant increases in *Mycobacterium* (*P =* 0.0044, FDR 0.0133).

## Discussion: What do these initial studies tell us?

These are clearly early days in the field of LC microbiomics but there appears to be some commonality in the bacterial genera which are changing in LC patients with different authors reporting changes in *Granulicatella* [43, 44], *Streptococcus* [42–44] and *Veillonella* [40, 41]. Granulicatella species are a normal component of the upper respiratory tract [45] but can also cause some serious infections such as endocarditis [46]. *Granulicatella* is part of the Lactobacilliales order within the Streptococcaceae (including the genus *Streptococcus*), which also includes the family Enterococcaceae where the *Enterococcus* genus is located [47]. Within this genus, we also observed the change in sputum from patients with LC compared to patients referred to a respiratory clinic but proved not to have cancer^−^. Therefore, it could be suggested that there seems to be some similarity in microbial responses to LC. *Streptococcus, Enterococcus* and *Granulicatella* are facultative anaerobes and the detection of *Veillonella* as an obligate anaerobe would argue that LC is associated with a switch to anaerobic fermentative respiration. In this context, it is relevant that the fermentative facultative anaerobe *Lactobacillus* is significantly increased in both COPD and LC patients where biofilm formation is prominent. However, the specificity that we have seen with *Granulicatella* would argue a specific microbiomic response to LC. *Granulicatella* is considered to be an example of nutritionally variant Streptococci as it requires such as pyridoxal (a form of vitamin B6) or L-cysteine to be supplemented to media in order to grow in the laboratory [46]. Our metagenomics strategy suggested functional changes in the LC microbiome which could be relevant to the increases in *Granulicatella*. In particular, it indicated increases in the microbiome's capacity to metabolise putrescine and its polyamine products [44]. This tallied with the increases in putrescine in LC patients detected in our parallel metabolomic study [48]. As polyamine and pyridoxal metabolism can be intrinsically linked, it may be that the increased abundance of *Granulicatella* could be a response to metabolites in these pathways [49]. Deregulation of putrescine and polyamines, which influence the cell cycle, has been noted in several cancers; including lung [50]. Therefore, *Granulicatella* could be responding to LC-linked increases in these metabolites.

Such considerations suggest that the microbiome reflects changes in the cancerous lung environment, but a causative role cannot be ruled out. The various mechanisms through which the microbiome could be causative in LC have been reviewed by Mao *et al* [38] and include inflammatory and immune changes which could reflect microbiological dysbiosis so that pathogens begin to predominate. In this context, the increases in genera with important pathogens, *Streptococcus* and *Mycobacterium* (Figure 1)*,* are relevant but these changes have not been observed in all studies. Here, comparisons with COPD may be informative where associations with a high prevalence of *P. aeruginosa*, *H. influenzae* and *S. pneumoniae* indicate an unequivocal role for pathogens [4, 16].

Taking all of our points together, there is a clear requirement for a larger scale assessment of microbiome changes in LC. Such studies should be sensitive to the heterogeneity of LC in terms of histology and stage and either focus on particular examples or be large enough to have the statistical power to allow valid conclusions to be made. A single sampling technique should be used, which although may be better done using BALF, restricts the number of samples which can be taken due to the invasive nature of this technique, and as such sputum samples may be a better alternative allowing larger numbers of samples to be obtained. Sputum also appears to retain the features of the lower respiratory tract. Finally, a full metagenomic strategy rather than 16S rRNA gene amplicon sequencing would allow functional changes in the microbiome to be revealed. Ideally, these could be supplemented with metatranscriptomic, metaproteomic or metabolomic assessments of samples so that these functional changes can be related to the wider lung environment. The challenge is considerable, but it does offer the possibility of yielding new insights into lung carcinogenesis, new biomarkers and possibly chemotherapeutic drug metabolism.

## Conclusions

The lung microbiome is now a well-established feature of many types of respiratory diseases. Preliminary microbiome analyses have indicated some changes in genera which include pathogens that are significantly associated with LC. These could indicate a role for microbially associated pro-inflammatory or immunological events in LC. Equally, the microbial changes could reflect changes in the LC environment to which the microbiome is responding. Whatever the cause, specific microbial changes could represent specific biomarkers for LC presence and type. However, the paucity of the number of LC microbiomic studies, their sample sizes and the range of sampling techniques used prevent any firm conclusions being made. More and larger studies are required.

## Authors’ contributions

All the authors wrote and edited the review. The representation of previously published data was based on the work of SJSC. The Medlung study was led by PDL and KL.

## Conflicts of interest

The authors declare that there are no conflicts of interest.
